# Editorial Comment on the Special Issue Discussing COVID-19 and Thrombosis, Second Edition

**DOI:** 10.3390/v15091922

**Published:** 2023-09-14

**Authors:** Pierpaolo Di Micco, Alessandro Perrella, Giuseppe Camporese

**Affiliations:** 1 AFO Medicina, P.O. Santa Maria delle Grazie, Pozzuoli, ASL Napoli2 nord, 80076 Napoli, CAP, Italy; 2 Department of Infectious Disease, PO Cotugno, AO dei Colli, 80131 Naples, CAP, Italy; alessandro.perrella@ospedalideicolli.it; 3 Department of Internal Medicine, General Medicine Unit, Thrombotic and Haemorrhagic Disorders Unit, University Hospital of Padua, 35128 Padua, CAP, Italy; giuseppe.camporese@aopd.veneto.it

The recent SARS-CoV-2 pandemic is ending after over three years, and the efforts of physicians in the daily clinical management of infection in inpatients and outpatients and vaccination campaigns allowed to medical experts to understand all possible scientific aspects of COVID-19.

One relevant clinical aspect has been COVID-19’s association with thrombotic complications, in particular pulmonary embolism. For this reason, a second edition of a Special Issue discussing the relationship between COVID-19 and thrombosis has been created, having a similar success among scholars to that of the first edition.

Of course, with increased experience in both scientific and clinical issues, topics discussed in the second edition of this Special Issue have focused not only on thrombotic events of the respiratory tract, but also those of the cerebral system.

COVID-19 clinical management practices changed in the most recent waves not only because the vaccination campaign reduced the number of severe COVID-19 cases, but also because the most recent viral variants have been less aggressive than the first variants. [1] Therefore, the triage system was also changed to reflect the presence of this anamnestic information [1]. Yet, for patients who also experienced COVID-19-associated thrombosis in the respiratory tract, clinical management differed based on information regarding the type of prophylaxis adopted and the localization of thrombus. From an anatomical point of view, great differences regarding type of antithrombotic treatment and related outcomes were noted during the latter waves of pandemic among patients with a typical pulmonary embolism and in situ pulmonal arterial thrombosis [2]. The roles played by prolonged inflammation and bacterial overlapping represented a further difference between these types of thrombosis [2]. Rare cases of pulmonary embolism in patients taking anticoagulation for other clinical reasons were noted, and they led to an aggressive course of disease [3]. The most recent viral variants showed a less aggressive course in adult, but this trend was not always recorded in children; thus, according to data regarding pulmonary embolism in children, an increased rate of this condition was detected in children who experienced COVID-19 [4].

The prolonged state of inflammation present since the first phases of SARS-CoV-2 infection [5] induces dysfunctions after viral clearance, and it is responsible of several coagulation-based abnormalities in patients with long COVID [5], especially in the presence of thrombophilia [6].

As during vaccination campaigns, an increased rate of cerebral venous thrombosis was found, during waves after the vaccination campaign began, particular attention was given to cerebral thrombosis, and COVID-19 was considered by specialists to be the most common acquired condition associated with cerebrovascular disease during the pandemic [7]. The alteration of haemostasis due to ACE dysregulation may induce a prothrombotic state that is dangerous in frail patients [8]. A rare case of ischaemic stroke occurred after the relapse of COVID-19 and administration of monoclonal antibodies toward SARS-CoV-2 was reported by Lobasso et al. [9], though as far as we know, it was a rare case of cerebral venous thrombosis that occurred in a young non-vaccinated woman [10].

Therefore, we can conclude that a new spectrum of information appeared in this second edition of this Special Issue related to COVID-19 and thrombosis, leading scholars to also consider COVID-19 to be a trigger risk factor of thrombotic complications that may appear during acute and subacute phases of infection, as well as during cases of long COVID. The relevant clinical experience gained during the pandemic due to *coronaviridae* may also be useful for future viral outbreaks.
